# In Vitro Anti-Inflammatory Activity in Arthritic Synoviocytes of *A. brachypoda* Root Extracts and Its Unusual Dimeric Flavonoids

**DOI:** 10.3390/molecules25215219

**Published:** 2020-11-09

**Authors:** Carlota Salgado, Hugo Morin, Nayara Coriolano de Aquino, Laurence Neff, Cláudia Quintino da Rocha, Wagner Vilegas, Laurence Marcourt, Jean-Luc Wolfender, Olivier Jordan, Emerson Ferreira Queiroz, Eric Allémann

**Affiliations:** 1 School of Pharmaceutical Sciences, University of Geneva, 1211 Geneva-4, Switzerland; carlota.salgado@unige.ch (C.S.); hugo.morin@unige.ch (H.M.); laurence.neff@unige.ch (L.N.); laurence.marcourt@unige.ch (L.M.); jean-luc.wolfender@unige.ch (J.-L.W.); olivier.jordan@unige.ch (O.J.); emerson.ferreira@unige.ch (E.F.Q.); 2 Institute of Pharmaceutical Sciences of Western Switzerland, University of Geneva, 1211 Geneva-4, Switzerland; 3 Departamento de Química Orgânica e Inorgânica, Universidade Federal do Ceará, 60450-765 Fortaleza-CE, Brazil; nayaracoriolano@hotmail.com; 4 Laboratório de Produtos Naturais, Centro de Ciência Exatas e Tecnologia, Departamento de Química, 65080-805 São Luís-MA, Brazil; claudiarocha3@yahoo.com.br; 5 Experimental Campus of the Paulista Coast, UNESP-São Paulo State University, 11330-900 São Vicente-SP, Brazil; vilegasw@clp.unesp.br

**Keywords:** *Arrabidaea brachypoda*, flavonoids, anti-inflammatory activity, osteoarthritis, high-speed counter current chromatography, mass-spectrometric quantification

## Abstract

*Arrabidaea brachypoda* is a plant commonly used for the treatment of kidney stones, arthritis and pain in traditional Brazilian medicine. Different in vitro and in vivo activities, ranging from antinociceptive to anti-*Trypanosoma cruzi*, have been reported for the dichloromethane root extract of *Arrabidaea brachypoda* (DCMAB) and isolated compounds. This work aimed to assess the in vitro anti-inflammatory activity in arthritic synoviocytes of the DCMAB, the hydroethanolic extract (HEAB) and three dimeric flavonoids isolated from the DCMAB. These compounds, brachydin A (**1**), B (**2**) and C (**3**), were isolated both by medium pressure liquid and high-speed counter current chromatography. Their quantification was performed by mass spectrometry on both DCMAB and HEAB. IL-1β activated human fibroblast-like synoviocytes were incubated with both extracts and isolated compounds to determine the levels of pro-inflammatory cytokine IL-6 by enzyme-linked immunosorbent assay (ELISA). DCMAB inhibited 30% of IL-6 release at 25 µg/mL, when compared with controls while HEAB was inactive. IC_50_ values determined for **2** and **3** were 3-fold higher than **1**. The DCMAB activity seems to be linked to higher proportions of compounds **2** and **3** in this extract. These observations could thus explain the traditional use of *A. brachypoda* roots in the treatment of osteoarthritis.

## 1. Introduction

*Arrabidaea brachypoda* (D.C.) is a shrub native to the Brazilian region of Cerrado (neotropical savanna). It belongs to the Bignoniaceae family, which includes 120 genera and nearly 800 species of different plants scattered in tropical and subtropical regions worldwide [1]. Plants of *Arrabidaea* genus are known sources of C-glucosylxanthones, phenylpropanoids, flavonoids, anthocyanidins, allantoins, and triterpenes [2,3,4]. These molecules are linked to the astringent, anti-inflammatory, antimicrobial, antitumoral and wound healing properties that plants of this genus are known for in traditional medicine [5]. In Brazil, *Arrabidaea brachypoda* is known as “cervejinha do campo”, a decoction of its roots used to treat kidney stones and arthritic joints [6,7]. Different molecules have been isolated and identified from the hydroethanolic extract (HEAB) and dichloromethane extract (DCMAB). From this last extract, three isolated aglycones—brachydin A (**1**), brachydin B (**2**), and brachydin C (**3**)—have been identified. In the HEAB, among the 14 described molecules, these three dimeric flavonoids exist in their glucoronated form [8,9]. Antinociceptive, anti-inflammatory, anti-*Trypanosoma cruzi*, gastroprotective, antileishmanial, and antimicrobial activities have been recently reported in different in vitro and in vivo assays from both root extracts and isolated compounds [8,9,10,11,12,13,14,15]. In these previous studies, different oral and/or topical administration setups were explored. Overall, studies reporting on anti-inflammatory and antinociceptive activities of *A. brachypoda* have focused on general mechanisms of pain and inflammation [10,12]. In this study, we aimed to test the in vitro anti-inflammatory activity of *A. brachypoda* root extracts and three isolated dimeric flavonoids in the context of osteoarthritis (OA). This chronic disease has worldwide incidence, in particular in the aging population and is considered the most common form of arthritis. Characterized by chronic pain and inflammation, articular cartilage degeneration, and structural changes of whole joints, OA represents the main cause of physical disability and a great health economic burden [16,17,18,19]. Presently, treatment options are primarily based on non-pharmacological and symptom management approaches. There is a need for long-acting, targeted local anti-inflammatory drug products, in order to tackle the main OA complications: pain and inflammation [20,21]. Human fibroblast-like synoviocytes activated for OA-like inflammation were used as the in vitro cellular model. Interleukin-6 (IL-6) is a ubiquitous pro-inflammatory cytokine of acute inflammation, particularly involved in the synovial hypertrophy, as well as an established therapeutic target for arthritis. Therefore, IL-6 was selected as the molecular marker of inflammation in this study [21]. Additionally, the isolated compounds were quantified in both HEAB and DCMAB to establish a link between the chemical composition of the extracts and anti-inflammatory properties of their individual constituents.

## 2. Results and Discussion

### 2.1. Isolation of Compounds ***1***–***3*** from the DCMAB of A. brachypoda

**Figure 1:**
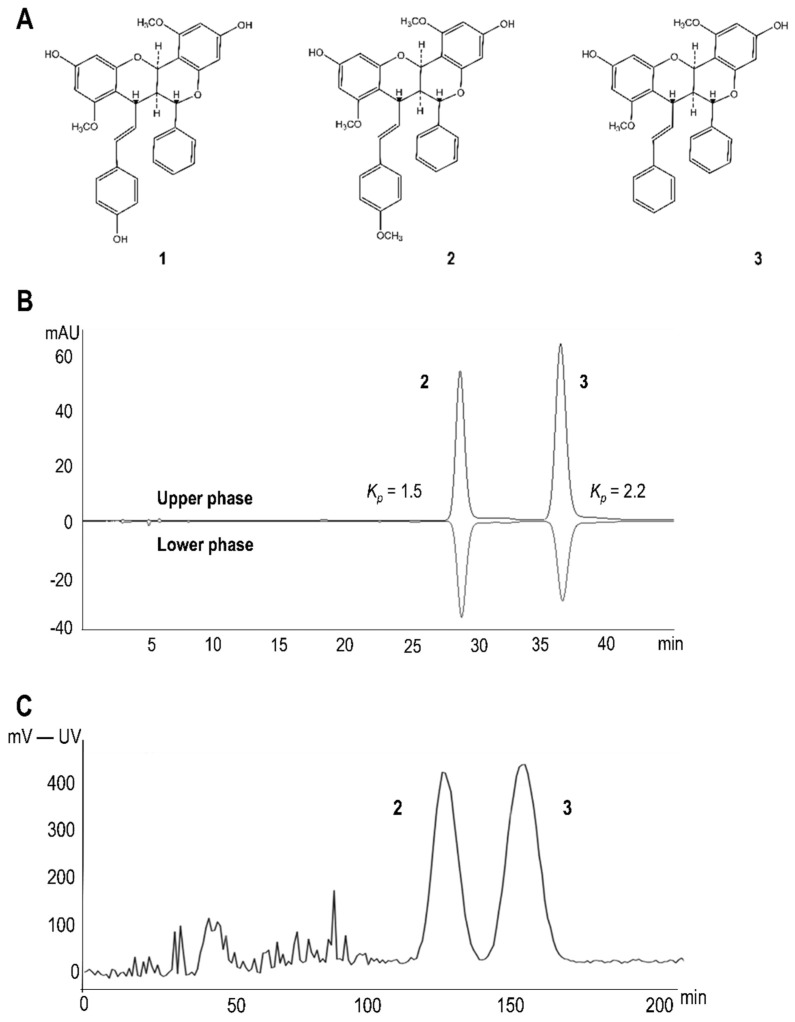
(**A**) Structures of brachydin A (**1**), brachydin B (**2**), and brachydin C (**3**) isolated from the dichloromethane extract (DCMAB) of *A. brachypoda* roots (**B**) Determination of coefficient of partition *Kp* by high-performance liquid chromatography with photodiode array detection (HPLC-PDA) of the solvent mixture P: methanol/ethyl acetate/hexane/water (6:5:6:5; *v/v/v/v*) of the ARIZONA system [23]. (**C**) High-speed counter current chromatography (HSCCC)-UV chromatogram at 280 nm, with clear separation of compounds **2** and **3**.

The DCMAB of *A. brachypoda* was obtained according to the protocol described in our previous study [8]. The high-performance liquid chromatography with photodiode array detection (HPLC-PDA) analysis revealed three major compounds (Figure 1) which were previously characterized as three unusual dimeric flavonoids named brachydin A (**1**), brachydin B (**2**), and brachydin C (**3**) [8]. To study the anti-inflammatory properties of these molecules, DCMAB was purified at large scale. As a first step, separation conditions were optimized at HPLC-PDA analytical scale (Figure S1, Supplementary Material) and transferred to medium pressure liquid chromatography (MPLC) at a semi-preparative scale, using a gradient transfer method. This yielded 4 g of brachydin A (compound **1**; Figure 1A) in a single step. Brachydin B (**2**) and brachydin C (**3**) were obtained in a mixture (4.9 g) (see Section 3.2.3), mostly due to a column overload, and were further purified by high-speed counter current chromatography (HSCCC). The solvent system was determined using the ARIZONA solvent system approach. The separation was carried out successfully as a result of the different *Kp* determined (compounds **2** (*Kp* = 1.5) and **3** (*Kp* = 2.2) (see Section 3.2.3). A full recovery of pure compound **2** (1.4 g) and **3** (3.2 g) (Figure 1C) resulted, as HSCCC is a support-free liquid-liquid partition chromatography technique [22]. According to the literature, this is the first time this technique has been described for the isolation of pure compounds from *A. brachypoda* root extracts.

### 2.2. Quantification of Compounds ***1***–***3*** from A. brachypoda Root Extracts by UHPLC-MS/MS

**Figure 2:**
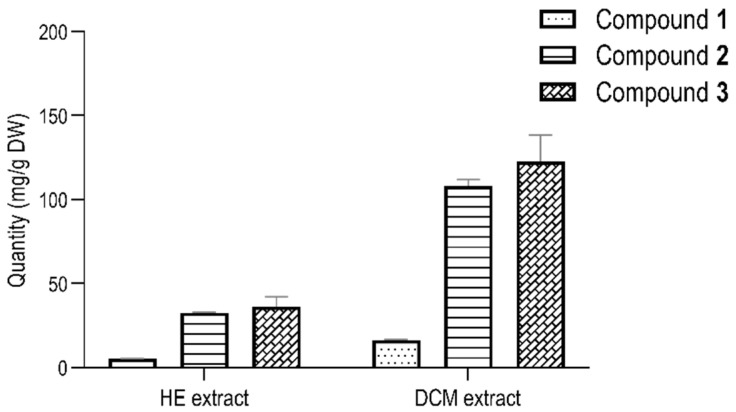
Concentration (mg/g DW) of compounds **1**–**3** in hydroethanolic extract (HEAB) and dichloromethane extract (DCMAB), respectively. Bars correspond to mean values ± S.D.; *n* = 3.

The amounts of compounds **1**, **2**, and **3** in the HEAB and DCMAB were determined by ultrahigh pressure liquid chromatography coupled to mass spectrometer (UHPLC-MS/MS) analysis, in the multiple reaction monitoring (MRM) mode. The MRM parameters of each analyte were optimized to increase sensitivity for a specific transition from mass spectrum^2^ (MS^2^) reported data (Table S1, Supplementary Material) [8]. The range of the calibration curves was estimated for each compound and was set to 31–500 ng/mL. This yielded five data point calibration curves with r^2^ > 0.99 (Table S2, Supplementary Material). The results obtained from the quantitative analysis are displayed in Figure 2. The total content of compounds **1**–**3** was 3.35-fold higher in DCMAB than in HEAB. This was expected, since these compounds are primarily occurring as glucoronated derivatives in HEAB [8], and these are not detected by MRM. Compound **3** was the most abundant in both DCMAB (123 mg/g (DW: dry weight) and HEAB (36 mg/g (DW)). Compounds **1** and **2** were present at 16 and 108 mg/g (DW) in DCMAB, respectively. Lower levels of **1** (5 mg/g) and **2** (33 mg/g (DW)) were found in HEAB. These results quantify, for the first time, the amounts of the three isolated brachydins in both extracts.

### 2.3. In Vitro Cytotoxicity and Anti-Inflammatory Bioactivity of Extracts and Isolated Compounds

#### 2.3.1. *Arrabidaea bachypoda* Extracts (HEAB and DCMAB)

**Figure 3:**
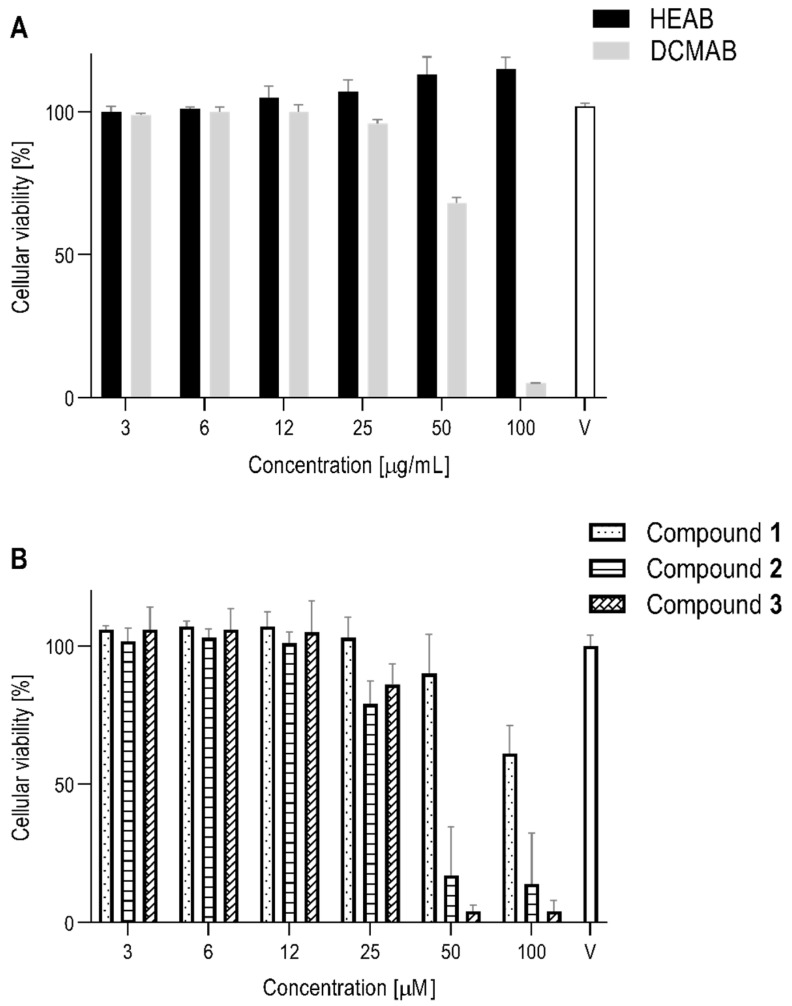
The cellular viability of human fibroblast like synoviocytes (HFLS) incubated with all tested compounds at increasing concentrations, after 24 h. Root extracts (**A**) and isolated compounds from dichloromethane extract (DCMAB) (**B**). Bars correspond to mean values ± S.D.; *n* = 6; V = vehicle, 0.01% dimethyl sulfoxide (DMSO). Additional scatter plot with individual data in Supplementary Material, Figure S4.

**Figure 4:**
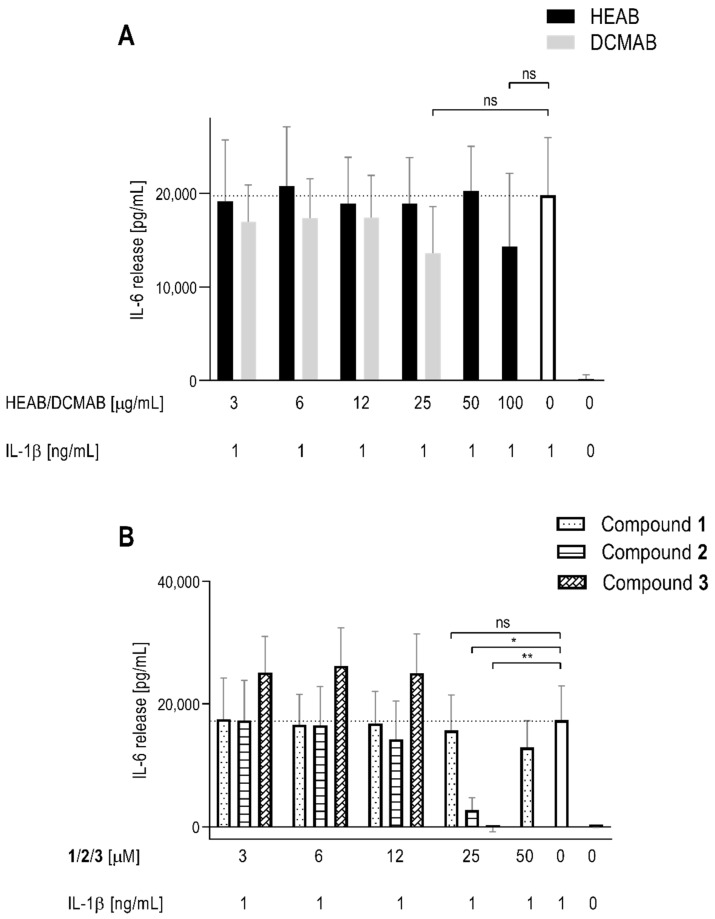
Release of pro-inflammatory cytokine IL-6 from IL-1β activated HFLS incubated with root extracts (**A**) and isolated compounds **1**–**3** (**B**), after 24 h. Bars correspond to mean values ± S.D.; *n* = 6. * *p* < 0.05, ** *p* < 0.009 and ns = no significance. Dotted lines represent cut-off of maximum IL-6 release by Il-1β stimulation.

The cytotoxicity of both HEAB and DCMAB was assessed; results are shown in Figure 3A (and corresponding scatter plot in Figure S4). Human fibroblast like synoviocytes (HFLS) were viable after 24 h incubation, with tested concentrations ranging from 3 to 100 µg/mL of HEAB. Conversely, at 50 μg/mL and 100 μg/mL of DCMAB, HFLS presented viability values of 68% and 5%, respectively, compared to controls (Figure 3A). Consequently, these two top concentrations were not considered in the bioactivity assay, a sandwich enzyme-linked immunosorbent assay (ELISA) (Figure 4A), due to toxicity. At the highest dose tested (100 μg/mL), HEAB showed a 30% inhibition of IL-6 release. DCMAB yielded a similar inhibitory effect at 25 μg/mL (31% inhibition; Table 1), the highest concentration inducing no cytotoxicity. Both inhibition effects were non-significant (*p* value 0.27 and 0.19, respectively) when compared to non-treated control: interleukin-1 beta (IL-1 β). In line with these results, IC_50_ calculations (Table 1) show that an anti-inflammatory effect occurs only above 100 µg/mL and 31 µg/mL for HEAB and DCMAB, respectively.

#### 2.3.2. Isolated Brachydins from DCMAB (**1**–**3**)

The three isolated compounds **1**, **2**, and **3**, were also tested for cytotoxicity (WST-1) and anti-inflammatory activity (ELISA) and showed different activities (Figure 3B, Figure S4 and Figure 4B). Compound **1** was only cytotoxic at 100 µM, whereas compounds **2** and **3** induced a loss of viability at 50 µM, with no evident effect of dose increase, unlike what was previously described for DCMAB in terms of cytotoxicity (Figure 3). For this reason, the IL-6 ELISA assay was performed using supernatants recovered after incubation with HFLS, up to 50 µM for **1** and only up to 25 µM for **2** and **3** (Figure 4B). At the highest concentration with remaining favorable viability (25 µM), both **2** and **3** significantly decreased IL-6 release when compared to the activation control IL-1β (80% and 94% inhibition, respectively (Table 1). Compound **1** showed a non-significant inhibition of IL-6 release at 25 µM and 50 µM (10% and 24%, respectively; Figure 4B and Figure S5, Supplementary Material). In Table 1, IC_50_ values (individual plots represented in Supplementary Material, Figure S6) confirmed the increased anti-inflammatory effect of **2** (17 µM) and **3** (19 µM), in comparison to **1** (62 µM). These different outcomes between compounds could be linked to the fact that both compounds **2** and **3** are of higher structural similarity between each other than compound **1**, that exhibits higher polarity influencing effects. Regarding cell viability of HFLS, HEAB and compound **1** presented the least cytotoxicity and DCMAB, compounds **2** and **3**, showed higher cytotoxicity. This is expected, since these are the major two components of this extract, as above mentioned (see Section 2.2). In order to compare the bioactivity results between the tested compounds, IC_50_ results in µM (isolated compounds) were transformed into µg/mL to match those of the extracts, that represent mixtures of several compounds (Table 1). In terms of the anti-inflammatory effect, results are in line with those of cytotoxicity. HEAB (IC_50_ > 100 µg/mL) was inactive in decreasing release of pro-inflammatory cytokine IL-6 in HFLS. According to what was previously reported for HEAB [9,10,11], this extract is comprised by the dimeric flavonoid aglycones (**1**–**3**) and their various glucoronated derivatives [9]. The glucoronated derivatives appear in higher amount than the corresponding aglycones. In an HFLS in vitro setting, the metabolic pathways that hydrolyze the glucoronated forms into simple aglycones (as it would in the acidic pH of the stomach) do not occur and the compounds are absorbed as is [24]. Based on such considerations, the lack of activity of HEAB can be thus correlated to the low amounts of **1**, **2**, and **3** (Figure 2) and the glucoronated derivatives themselves, inactive in this experimental setup. This is also the reasoning as to why all current studies and evaluations of this extract in vivo occur after *per os* administration and not local [8,9,10,12]. DCMAB on the other hand, only contains the three isolated brachydins A, B, and C in their non-glucoronated forms. Such extract is thus more interesting to explore in this experimental setup and when seeking local administration of anti-inflammatory treatments for OA. Albeit through unknown pathways, this study is the first analyzing the anti-inflammatory activity in the specific context of OA. Additionally, it compares effects and quantifies the presence of isolated compounds in both *A. brachypoda* extracts in order to assess their potential as OA therapeutic alternatives. Compound **1** exhibited similar anti-inflammatory activity (IC_50_) when compared to DCMAB (33 µg/mL and 31 µg/mL, respectively), whereas compounds **2** (9 µg/mL) and **3** (10 µg/mL) showed a 3-fold higher activity. This suggests that the extracts activity results mainly from the presence of the two latter compounds. Taking into account the amounts of each compound **1**–**3** in the DCMAB (Table 1), the cumulative IC_50_ of each individual isolated compound (27 µg/mL) does not greatly differ from DCMAB alone—31 µg/mL. Based on these results, no investigation on synergistic effect was pursued. Differences between the anti-inflammatory activities between the three compounds potentially stem from the higher polarity of compound **1**, in comparison to **2** and **3**.

## 3. Materials and Methods

### 3.1. Materials

#### 3.1.1. General Experimental Procedures

All analytical HPLC-PDA analyses were performed using an Agilent Technologies 1260 Infinity system equipped with a photodiode array detector (Agilent Technologies, Santa Clara, CA, USA). Preparative medium pressure liquid chromatography (MPLC) was performed using a system equipped with a C-605 module pump, C-640 UV detector, and C-684 fraction collector all from Büchi (Flawil, Switzerland). The system was controlled by the Sepacore Control software (Büchi AG, Flawil, Switzerland). A coil connected to a resistance was used to control the MPLC column temperature. The column (460 mm × 49 mm i.d.) was packed with ZEOprep^®^ C18 as the stationary phase (ZEOprep^®^ C18, 15–25 µm; Zeochem, Uetikon am See, Switzerland). Nuclear magnetic resonance (NMR) spectroscopic data were recorded on a Bruker Avance III HD 600 MHz NMR spectrometer equipped with a QCI 5 mm Cryoprobe and a SampleJet automated sample changer (Bruker BioSpin, Rheinstetten, Germany). Chemical shifts were reported in parts per million (δ) using the CD_3_OD residual signal (δH 3; δC 49) as internal standards for 1H and 13C NMR and coupling constants (J) were reported in hertz. High-speed counter-current chromatography (HSCCC) coupled to UV was performed on a Tauto TBE-300B instrument (Tauto Biotech, Shangai, China) equipped with two LC10AD HPLC pumps (Shimadzu, Kyoto, Japan), a 20 mL injection loop, a Knauer K 2501 UV detector (Berlin, Germany) and a C-684 fraction collector from Büchi (Flawil, Switzerland). Solvents used for extraction (methanol, ethyl acetate, hexane) were all of analytical grade. Solvents used in the quantification of the isolated brachydins: methanol, formic acid, acetonitrile and water were of LC-MS grade. All solvents were purchased from Sigma-Aldrich (St. Louis, MO, USA). Milli-Q^®^ water from Merck (Burlington, MA, USA) was used throughout this study. All other chemical products were obtained from Sigma-Aldrich (St. Louis, MO, USA). For the in vitro anti-inflammatory assays, all media solutions and ELISA kits were purchased from Invitrogen (Carlsbad, CA, USA). Fetal bovine serum was purchased from Eurobio (Les Ulis, France). Interleukin 1 beta (IL-1β) was obtained from R&D Systems (Bio-Techne, Abingdon, UK).

#### 3.1.2. Plant Material

Roots of *Arrabidaea brachypoda* were collected in April 2010 from the Sant’Ana da Serra farm in João Pinheiro, Minas Gerais, Brazil. The plant was identified at the ICEB of José Badine Herbarium of the Federal University of Ouro Preto by Prof. Maria Cristina Teixeira Braga Messias. A voucher specimen (#17935) was deposited at the Herbarium of the Federal University of Ouro Preto, Brazil. The plant was collected in accordance with Brazilian authorities (SISGEN #A451DE4).

### 3.2. Extraction and Isolation of Brachydins A *(**1**)*, B *(**2**)* and C *(**3**)*

#### 3.2.1. Plant Extraction

Both HEAB and DCMAB of the roots of *Arrabidaea brachypoda* were obtained from our previous study [9]. These extracts were stored and protected from light at −20 °C.

#### 3.2.2. HPLC-PDA Analysis

The DCMAB of *A. brachypoda* (Figure S1, Supplementary Material), the MPLC and HSCCC fractions were analyzed by HPLC-PDA using a Waters X-Bridge C18 column (250 mm × 4.6 mm i.d., 5 μm; Waters, Milford, MA, USA) equipped with a Waters C18 pre-column cartridge holder (10 mm × 2.1 mm i.d.). Water (A) and methanol (B), both containing 0.1% of formic acid (FA) were used as the solvent system. The column was equilibrated with 5% of B for 15 min. The separation was performed in gradient mode, as follows: 5 to 100% of B in 60 min, and 100% of B for 10 min. Flow rate 1 mL/min; injection volume 10 μL; sample concentration 10 mg/mL in methanol. The UV absorbance was measured at 254 nm and the UV-PDA spectra were recorded between 190 and 600 nm (step 2 nm).

#### 3.2.3. Isolation of Brachydins A (**1**), B (**2**), and C (**3**)

DCMAB was fractioned by MPLC, following a previously published protocol [25]. Fractionation conditions were optimized on an HPLC column (see Section 3.1.1 and Section 3.2.2) using the same stationary phase and an acidic (0.1% FA) water (A) and methanol (B) gradient. Solvent system gradient went as follows: isocratic 5% B in 56 min, 5 to 38% B in 5.5 h, isocratic 38% B in 4.7 h, 38 to 62% B in 3.2 h, 62 to 100% B in 9 min and isocratic 100% B during 1.5 h. A dry load injection of the sample was performed by mixing 7 g of the dichloromethane extract with 35 g of Zeoprep^®^ C18 (40–63 µm). The dry-load cell (11.5 cm × 2.7 cm i.d.) was subsequently connected between the pumps and the MPLC column. The flow rate was set to 20 mL/min and UV absorbance was monitored at 280 nm. MPLC separation yielded 90 fractions of 250 mL, which were analyzed by ultrahigh pressure liquid chromatography coupled to an UV detector (UHPLC-UV). This approach yielded gram amounts of compound **1**. Fractions containing pure compounds were combined, dried, analyzed by nuclear magnetic resonance (NMR) and properly stored. Other MPLC fractions containing mixtures of compounds **2** and **3** were combined and subjected to purification by HSCCC coupled to a UV detector. The ARIZONA solvent system approach was applied to perform the separation of compounds **2** and **3**. This approach is based on the use of 23 solvent mixture compositions of methanol/ethyl acetate/hexane/water. The mixtures are labeled with letters from the alphabet from A to Z (except E, I, and O) [23]. To determine the best-suited ARIZONA solvent mixture, the partition coefficient (*Kp*) of each compound was determined. As so, few milligrams of the fraction containing **2** and **3** were solubilized in a specific mix of the ARIZONA system. Upper and lower phases of each mixture of solvents were separated and analyzed by HPLC-UV. The UV area peak of each compound was used to determine its coefficient of partition (*Kp*) according to the equation in Figure S2 (Supplementary Material). The best results for complete separation of compounds **2** and **3** were obtained by solvent mixture P-methanol/ethyl acetate/hexane/water (6:5:6:5; *v/v/v/v*). The coil was first filled with the two phases (upper and lower, 1:1) and rotation was set to 1000 rpm. Lower phase was then pumped into the column at a flow rate of 3 mL/min using the head-to-tail mode (mobile phase = lower phase; stationary phase = upper phase) with rotation set at 8000 rpm. After equilibrium between the two phases, 500 mg of sample in 20 mL of upper and lower phase solution (1:1) were injected. Four injections were performed. A total of 35 fractions (5 ml each) were obtained for each injection. The fractions were combined according to the chemical composition determined by HPLC-PDA analysis, dried, analyzed by NMR, and properly stored.

### 3.3. Quantification of Compounds ***1***–***3*** from A. brachypoda Root Extracts by UHPLC-MS/MS

Sample preparation was carried out by dissolving the dried HEAB and DCMAB from *Arrabidaea brachypoda* roots in 100% methanol. The final concentration of the samples was 10 µg/mL, and three technical replicates were used for quantitative analysis. The amounts of compounds **1**, **2**, and **3** in the extracts were quantified on a QTRAP 4000 quadrupole linear ion-trap mass spectrometer (Sciex, Darmstadt, Germany) with an ESI interface operating in positive ionization mode. UHPLC was performed on an Acquity UPLC system (Waters, Milford, MA, USA) equipped with a Waters UPLC BEH C18 column (50 mm × 2.1 mm ID, 1.7 µm). The solvent system consisted of water: 0.1% FA for solvent A and acetonitrile: 0.1% FA for solvent B. The elution was performed in gradient mode at 40 °C (400 µL/min flow rate) with the following steps: 5 to 60% B in 4.2 min, 60 to 70% B in 1.3 min, 70 to 100% B in 1.5 min, maintaining B at 100% for 1 min and lastly, a reconditioning with 5% B for min. The autosampler compartment was set at 10 °C throughout the analysis and the injection volume was 5 µL. MS/MS quantitative analyses were performed in multiple reaction monitoring (MRM) mode. Source and gas parameters were as follows: curtain gas, 10 psi; collision gas, 10 psi; ionspray voltage, 3500 V; source temperature, 400 °C; ion source gas 1, 40 psi; ion source gas 2, 50 psi. Nitrogen was used as collision gas. MRM transitions and MRM parameters were all optimized via flow injection analysis (FIA) technique with a mix containing 10 ng/mL of pure **1**, **2**, and **3** analytes (Table S1, Supplementary Material). Quantification was achieved by constructing three separate external calibration curves from solutions of the isolated pure compounds. The calibration curve concentrations for active compounds were as follows: 500 ng/mL, 250 ng/mL, 125 ng/mL, 63 ng/mL, 31 ng/mL. As no analyte-free matrix was available, the displayed limits of quantification (LOQ) were evaluated only on each pure standard. A signal-to-noise ratio of 10 was considered as the approximate LOQ (Table S2, Supplementary Material).

### 3.4. In Vitro Anti-Inflammatory Bioactivity

#### 3.4.1. Human Fibroblast-like Synoviocytes (HFLS) Isolation and Culture

Hip synovial membrane was collected from three male adult patients with clinical osteoarthritis (OA) at the time of hip replacement surgery. This protocol was conducted under the approval of the local Ethics Committee (CCER, Geneva, Switzerland) (Authorization #2017-02234) and with informed and consenting patients. Once collected, tissue samples were processed, according to previously described protocols [26]. Briefly, samples were finely minced and digested for 3 h (37 °C, 5% CO_2_ incubation) in a 3 mg/mL collagenase IX-RPMI 1640 solution. After centrifugation (200× *g*) and supernatant removal, the resuspended pellet was cultured (37 °C, 5% CO_2_) in medium containing RPMI 1640, M199 (1:1), 1% penicillin/streptomycin (100 IU/mL:100 g/mL), 2 mM l-glutamine and 20% fetal bovine serum. After overnight culture, non-adherent cells were removed. At confluence, cells were trypsinized and passaged to 75 cm^2^ culture flasks (Corning, NY, USA) in complete medium containing 10% fetal bovine serum. A double control was performed to confirm presence of HFLS after isolation: visual morphology evaluation (Figure S3) and flow cytometry with synoviocyte specific surface markers (CD14^+^ and VCAM-1). A representative sample of all cell populations was found to have above 90% HFLS markers. All cellular assays were performed from passage 3 to 9. Experiments were conducted twice per donor (*n* = 6).

#### 3.4.2. In Vitro Cytotoxicity Assay and Bioactivity Assay with the Determination of IL-6 Release in HFLS

Confluent HFLS cells (25,000 cells/well) were treated in a 96-well plate with compounds **1**, **2**, and **3** (3 µM, 6 µM, 12 µM, 25 µM, 50 µM, and 100 µM), HEAB and DCMAB (increasing concentrations from 3 to 100 µg/mL), or control vehicle for 1 h. All tested compounds were dissolved in a dimethyl sulfoxide (DMSO) stock solution and further diluted in culture medium. Final concentration of DMSO in vehicle of tested concentrations was 0.01%. After 1 h incubation, cells were activated by addition of IL-1β (1 µg/mL) (R&D Systems, Bio-Techne, Abingdon, UK) with subsequent incubation for 23 h. All supernatants were kept at −80 °C, before further testing. Remaining adherent cells were tested for viability using the cell proliferation reagent WST-1 (Roche, Basel, Switzerland), according to the supplier instructions. Pro-inflammatory cytokine IL-6 release in cell supernatant was assessed by ELISA. For this, a human IL-6 ELISA kit from Invitrogen (ref #88-7066-88; Thermo-Fisher Scientific, Waltham, MA, USA) was used according to the manufacturer’s protocol. All samples were diluted 90 times. Experiments were conducted twice per donor (*n* = 6).

### 3.5. Half Maximal Inhibitory Concentration (IC_50_) Determination and Statistical Analysis

In this study, data are presented as mean values ± standard deviation (S.D.). All analyses were performed using GraphPad Prism 8.3 software. IC_50_ values were determined using a nonlinear fitting of a log (inhibitor concentration) vs response variable slope for each donor’s data set. Consequent values were then averaged. After confirming the data normal distribution and homogeneity of variances (Brown-Forsythe and Welch; Shapiro-Wilk, D’Agostino-Pearson and Anderson-Darling), statistical analysis was performed using a two-way ANOVA with a Bonferroni multiple comparisons test. Significance was determined at alpha level 0.05. Significance values represented are * *p* < 0.05, ** *p* < 0.009 and ns for non-significance.

## 4. Conclusions

*Arrabidaea brachypoda* is a plant commonly used in Brazilian traditional medicine to treat pain and inflammation related to arthritic joints. The effects of HEAB, DCMAB and three isolated dimeric flavonoids were assessed. In this study, specific in vitro anti-inflammatory activity was tested using primary human arthritic synoviocytes, in order to understand the mechanisms involved and potentially identify molecules of interest for local drug delivery in OA. The present study is the first comparing the anti-inflammatory effect and providing a quantification of the three isolated compounds from the DCMAB. Despite high cellular viability scores at tested concentrations, HEAB was not performant in terms of anti-inflammatory effect. Studies suggest that the acidic hydrolysis in the stomach is responsible for the releasing of aglycones deriving from glucoronated forms of not only brachydins A, B, and C, but also other molecules of the extract [8,24]. In this study setup however, hydrolysis does not take place, which explains the lack of activity of HEAB. Conversely, DCMAB showed an effect against the release of pro-inflammatory cytokine IL-6. Quantification of **1**, **2**, and **3** in this extract showed higher proportions of **2** and **3** compared to **1**. The bioactivity results are in line with these findings, where **1** alone is considerably less active than to **2** and **3** alone. Compound **3** presented as both the most effective and cytotoxic compound, the fact that it is the main component of DCMAB could account for the extract’s enhanced effect over both **1** and **2** and, partly, HEAB. Differences in polarity between compound **1** versus **2** and **3** can affect bioavailability and bioactivity. Compounds **2** and **3** seem to have a major role in the anti-inflammatory effects of DCMAB and, potentially, other aglycones present in HEAB deserve further exploring in terms of anti-inflammatory bioactivity. Additionally, further research of in vivo studies and potential drug delivery systems would enrich the understanding of the mechanisms underlying *A. brachypoda* roots and its treatment of arthritic joints.

## Figures and Tables

**Table 1:** IL-6 inhibitory activity and anti-inflammatory IC_50_ of all tested compounds.

Extracts		IC_50_ (µg/mL)		Inhibition of IL-6 Release at Highest non-Toxic Concentration (%) [25 µg/mL]		
HEAB		>100		2 ± 12		
DCMAB		31 ± 1		31 ± 8		
Compounds	Molecular Weight (g/mol)	IC_50_		Inhibition of IL-6 release at highest non-toxic concentration (%) [25 µM = 13 µg/mL *]	Compound in DCMAB (% of DW **)	Compound concentration in IC_50_ of DCMAB (µg/mL)
		(µg/mL)	(µM)			
**1**	524	33 ± 2	62 ± 2	10 ± 11	2	0.5
**2**	538	9 ± 3	17 ± 3	80 ± 9	10	3.4
**3**	508	10 ± 3	19 ± 3	94 ± 5	12	3.8

* Mean value for equivalence to 25 µM of Compound **1**, **2** and **3**. ** Dry Weight (DW).

## Supplementary Materials

The following are available online, Figure S1: Reverse phase HPLC-UV analysis at 254 nm, Figure S2: Coefficient of partition *Kp* equation, Figure S3: Human fibroblast like synoviocytes, Figure S4: Plot of individual values of cellular viability of extracts and isolated compounds, Figure S5: Inhibition percentage of IL-6 release normalized to 100% activation, Figure S6: IC_50_ calculations of extracts and isolated compounds, Table S1: Optimized MRM parameters for the quantification of active compounds by UHPLC-MS/MS, Table S2: Calibration curves parameters for active compounds and determination of the LOD and LOQ in ng/mL.
