# Supplementary material for: In Vitro Anti-Inflammatory Activity in Arthritic Synoviocytes of A. brachypoda Root Extracts and Its Unusual Dimeric Flavonoids
Source: Molecules. 2020 Nov 9;25(21):5219. doi: 10.3390/molecules25215219 (PMC7665123; doi:10.3390/molecules25215219)
Supplement: Supplementary file 1 [file molecules-25-05219-s001.pdf]

1. Supplementary Material

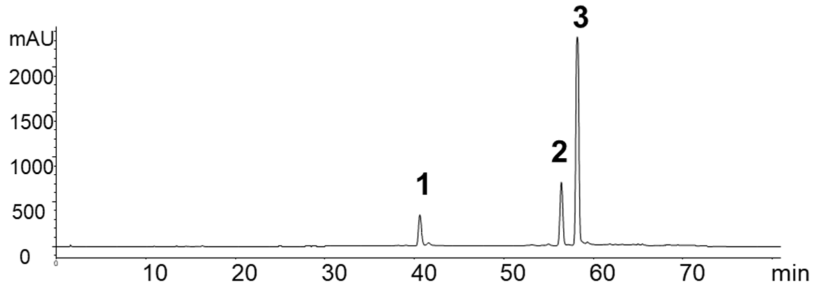

Figure S1. Reverse phase HPLC-UV analysis at 254 nm - optimized conditions for MPLC separation. Brachydin A (**1**), Brachydin B (**2**) and Brachydin C (**3**).

$$Kp = \frac{[S]_{\text{upper phase}}}{[S]_{\text{lower phase}}}$$

Figure S2. Coefficient of partition K_p equation (ARIZONA system).

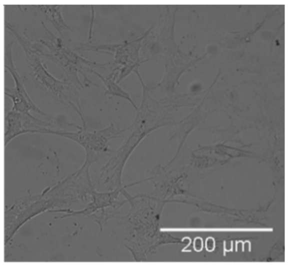

Figure S3. Human fibroblast like synoviocytes (optical microscope). Scale bar: 200 µm.

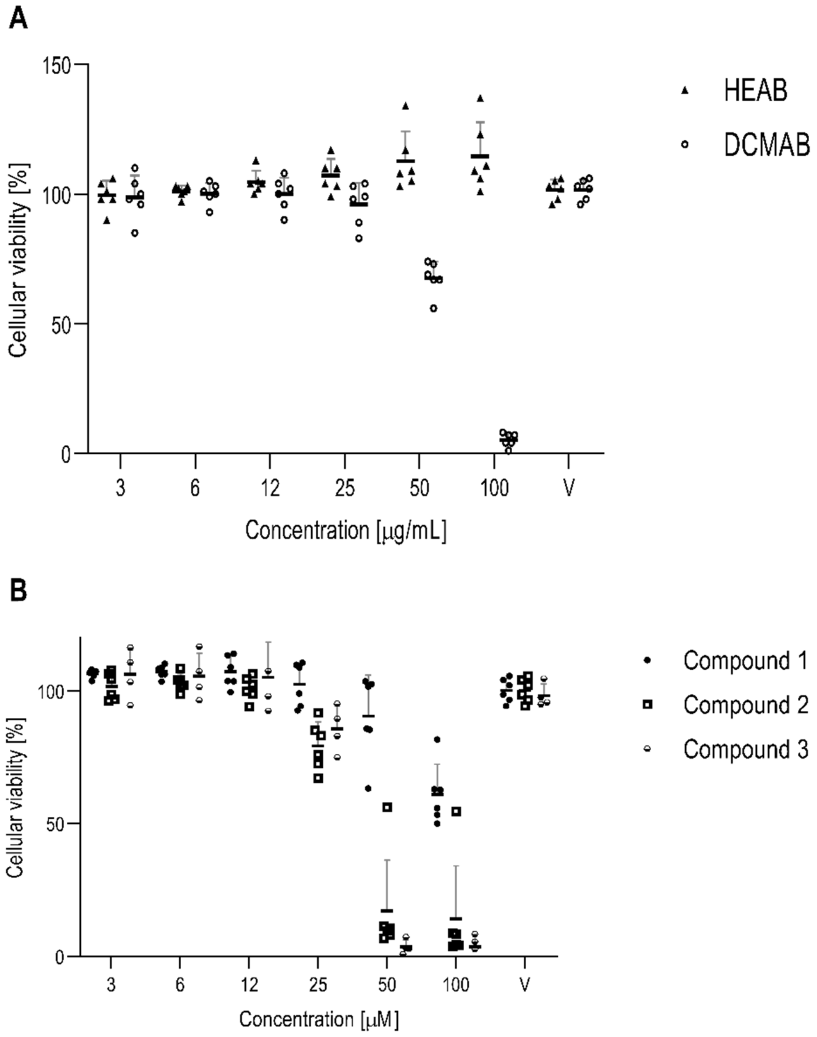

Figure S4. Scatter plot of Figure 3, showing cellular viability of human fibroblast like synoviocytes (HFLS) incubated with all tested compounds at increasing concentrations, after 24 h. Root extracts (**A**) and isolated compounds from dichloromethane extract (DCMAB) (**B**). Individual plotted values (n = 6) with mean values ± S.D.. V = vehicle, 0.01 % dimethyl sulfoxide (DMSO).

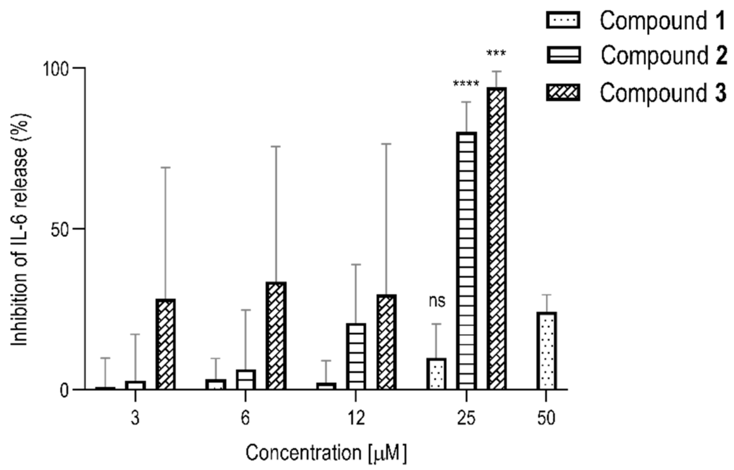

Figure S5. Inhibition percentage of IL-6 release normalized to 100 % activation, as a function of each compound concentration. Bars correspond to mean values ± S.D.; n = 6.**** p <0.0001 and *** p = 0.0007 and ns = no significance.

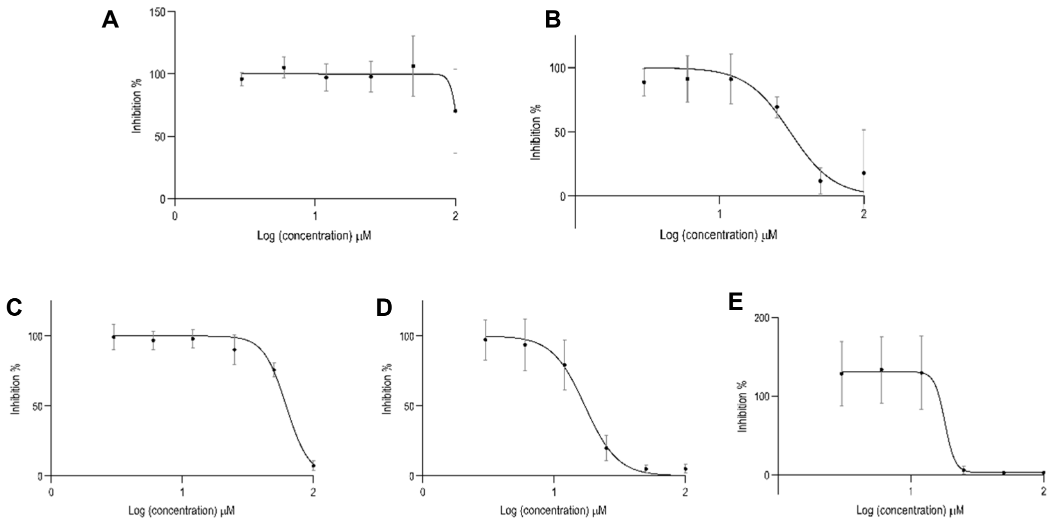

Figure S6. IC_{50} calculations of hydroethanolic extract (HEAB) (**A**), dichloromethane extract (DCMAB) (**B**), **1** (**C**), **2** (**D**) and **3** (**E**). Points correspond to mean values ± S.D.; n = 6.

Table S1. Optimized MRM parameters for the quantification of active compounds by UHPLC-MS/MS.

Compound	Precursor Ion	SRM transition	DP (V)	EP (V)	CE (eV)	CXP (V)	Dwell time (ms)
**1**	525	525 → 271	30	10	10	7	125
**2**	539	539 → 285					
**3**	509	509 → 255	80		50	12	

SRM, Selected reaction monitoring; DP, Declustering potential; EP, Entrance potential; CE, Collision energy; CXP, Collision cell exit.

Table S2. Calibration curves parameters for active compounds and determination of the LOD and LOQ in ng/mL.

Compound	Linear function	r^2	LOD (ng/mL)	LOQ (ng/mL)
**1**	$y = 816x + 6{,}06.10^3$	0.998	0.21	0.64
**2**	$y = 422x + 3{,}51.10^3$		0.47	1.41
**3**	$y = 122x + 639$		1.42	4.25

r^2, Correlation coefficient; LOD, Limit of Detection; LOQ, limit of quantification.
